# Predictions about the Cognitive Consequences of Language Switching on Executive Functioning Inspired by the Adaptive Control Hypothesis Fail More Often than Not

**DOI:** 10.3390/brainsci11091217

**Published:** 2021-09-15

**Authors:** Kenneth R. Paap, Lauren Mason, Regina Anders-Jefferson

**Affiliations:** 1 Department of Psychology, San Francisco State University, 1600 Holloway Avenue, San Francisco, CA 94019, USA; reginaa@sfsu.edu; 2 Department of Psychology, Tufts University, Medford, MA 02155, USA; Lauren.Mason@tufts.edu

**Keywords:** bilingualism, attention, adaptive control hypothesis, executive functions, language switching

## Abstract

The adaptive control hypothesis developed by Green and Abutalebi is the most influential theory of bilingual language control. The focus of this article is on the predictions that other researchers have derived based on the three different modes of interactional context described by the hypothesis. Foremost, that dual-language contexts should enhance domain-general executive functions more than single-language contexts. Several recent and ambitious behavioral tests of these predictions are reviewed. Although there was some evidence that dual-language contexts are associated with smaller switch costs, the evidence is inconsistent and there were no similar advantages for inhibitory control. The hypothesis also predicts neuroanatomical adaptations to the three types of interactional context. A careful evaluation of the relevant fMRI and ERP studies that take into account whether behavioral differences align with neuroscience differences and resolves valence ambiguities led to the conclusion that the neuroscience evidence for the hypothesis is, at best, inconsistent. The study also includes new analyses of two large-sample studies that enable the identification of relatively pure cases of single-language bilinguals, dual-language bilinguals, and dense-code switchers. Across nine different measures of executive functioning, the predicted advantage of the dual-language context never materialized. The hypotheses derived from the adaptive control hypothesis do not accurately predict behavioral performance on tests of executive functioning and do not advance our understanding as to what dimensions of bilingualism may lead to enhancements in specific components of executive functioning.

## 1. Introduction

The most cited article [1] in the *Journal of Cognitive Psychology* is Green and Abutalebi’s (2013) article titled *Language control in bilinguals: The adaptive control hypothesis* (ACH) with 942 Google-scholar citations in August 2021. The ACH proposed that different types or patterns of language switching required different types of cognitive control to manage the competition between a bilingual’s two languages. Three modes were distinguished. In the single language context (SLC), bilinguals tend to use one specific language in each context. For example, Spanish is used at home and English is used at the university. When entering a context, the competition between the languages is managed by activating the appropriate language schema which, in turn, inhibits the non-target language. As indicated in Green and Abutalebi’s Table 1, this recruits mechanisms (that are presumably domain-general) for goal maintenance, conflict monitoring, and interference suppression that should be strengthened compared to monolinguals. Bilinguals who frequently switch languages in the same context (especially when speaking to different addressees) also recruit goal maintenance, conflict monitoring, and interference suppression, but must additionally recruit selective response inhibition and task disengagement and engagement processes as they switch control from one language schema to the other. Frequent experience in this dual language context (DLC) is assumed to place more frequent demands on seven specific processes. As those processes strengthen, it should confer benefit on bilinguals who operate most of the time in DLC mode. The seven processes are: (1) goal maintenance, (2) conflict monitoring, (3) interference suppression, (4) salient cue detection, (5) selective response inhibition, (6) task engagement, and (7) task disengagement. To be more specific, Green and Abutalebi assume that DLC, compared to SLC, requires more (1) goal maintenance and (2) conflict monitoring, but that even SLC requires more goal maintenance and conflict monitoring than a monolingual speaker in a monolingual context. DLC also recruits processes (3) to (7), but SLC does not exceed a neutral baseline defined as the amount needed by a monolingual speaker in a monolingual context. Thus, all seven processes are used more in DLC than SLC and predict an advantage of DLC bilinguals over SLC bilinguals. These predicted differences are, of course, predicated on the assumption that these are general control-processes, not task specific and, in particular, not encapsulated within a language processing system—a possibility we have discussed in detail [2]. By logical extension, DLC bilinguals should also show advantages compared to monolinguals. 

The third mode involves dense code-switching (DCS) that was operationally defined within the ACH as mixing languages within an utterance or sentence. The critical assumption is that dense code-switching is best accomplished in an open control mode that eschews the need for inhibitory control. Thus, bilinguals who primarily engage in DCS have not honed domain-general inhibitory control to the same degree as DLC bilinguals and therefore, may perform less well on tests of general inhibitory control. They may even show inferior performance compared to SLC bilinguals. 

To clarify, the ACH is a theory of bilingual language control and not a theory of when bilingualism should lead to advantages in domain-general EF. As reviewed in an upcoming section, many bilingualism researchers have used the ACH as a framework for predicting when bilingual advantages should and should not occur. This approach has led to publications in highly selective journals in general cognitive psychology (e.g., Cognition, Journal of Experimental Psychology: General) and bilingualism (Bilingualism: Language and Cognition), and we want to alleviate concerns that our evaluation of the ACH may be biased or otherwise unfair. The preceding paragraphs suggested that the ACH predicts an advantage of DLC bilinguals over SLC bilinguals on nonverbal tests that require inhibitory control. However, earlier versions of this manuscript and a presentation at the annual meeting of the Psychonomics Society alerted us that some experts feel that Green and Abutalebi (2013) either did not make such predictions, that such predictions do not follow from the ACH, or that they are at least somewhat nonsensical. A sample concern expresses something like this: SLC, DLC, and DCS are contexts, not participant characteristics. They are not mutually exclusive categories. It is highly unlikely that bilingual individuals can be assigned to one context or another, because most bilinguals will engage in all these scenarios to some extent. 

There are certainly undeniable truths in these assertions, but Green and Abutalebi [1] did state: “*With respect to experimental tasks that tap specific component control processes adaptive effects should be evident in the analysis of reaction time distributions for conflict tasks such as the color-word Stroop task, Our analysis leads to the prediction that bilingual speakers in the dual-language context will be the more proficient in inhibition than those in the single-language or dense code-switching contexts*”, p. 522. What Green and Abutalebi appear to be saying is that IF the core assumptions of the ACH (i.e., that different interactional contexts demand different control processes and that the processes will adapt to these demands) are correct and if those adaptive effects are of sufficient magnitude and our measures have adequate precision, then the direction of those differences should favor bilinguals with DLC proclivities. 

The fact that the three contexts are not mutually-exclusive clearly creates a challenge in testing those predictions. In the review that follows, two approaches will be carefully examined. One is to derive a measure of DLC tendency (i.e., the degree to which a specific bilingual is immersed in dual-language interactions) and to use regression analyses to test for a positive relationship between DLC tendency and performance in tasks of domain-general EF. A second approach is to start with a large database of bilinguals and to use a priori criteria to select relatively “pure” cases of DLC, SLC, and DCS bilinguals, although conceding the prior point that genuine pure cases do not exist. Both of these approaches are fraught with the additional problems associated with the accuracy of self-reports describing the use of the languages one speaks. Indeed, a second important purpose of this paper is to carefully examine the strengths and weaknesses of the current tools and measures for investigating hypotheses about the relative advantages between different types of bilinguals or between bilinguals and monolinguals.

Another issue to address up front is the fact that the primary predictions of the ACH were not concerned with advantages in far transfer (i.e., to nonverbal tests of domain-general EF). Rather, many interesting predictions regarding bilingual control during speech production and comprehension were offered. Thus, when we conclude that the far-transfer predictions often fail, that does not detract from the value of the ACM in generating interesting research or the accuracy of its predictions regarding bilingual language processing in its own right. The latter is outside the scope of this paper. 

## 2. The Neuroscience of the ACH

### 2.1. The Neuroanatomy of Bilingualism

The ACH proposes a neural network for bilingual language control during bilingual speech production [1,3]. The underlying cognitive processes involve the intention to speak in a given language, selection of the target response, inhibition of words from the non-target language, and monitoring for intrusions. The network for control during a dual-language interaction includes the left prefrontal cortex, the anterior cingulate cortex, the caudate/putamen, the parietal lobule, the thalamus, and the right inferior frontal cortex. In a dual-language context, both languages can be active and to speak in the target language the speaker must maintain the goal, detect salient cues, control interference, and eventually inhibit responses with task engagement and disengagement. Because the essence of ACH is that language control processes adapt to the recurrent demands of the interactional context, a dual-language context should heavily recruit bilaterally the inferior frontal and parietal cortices, the ACC/pre-SMA, basal ganglia, and thalamus [3] and induce changes in the neuroanatomy compared to monolinguals or other types of bilinguals. Before reviewing the evidence, it is important to emphasize the cautionary notes piped by the Basque Center on Cognition Brain and Language (BCBL) [4]: *“Supporting evidence for an advantage should involve showing that these differences are accompanied by unambiguous behavioral data substantiating a cognitive gain.”* Duñabeitia and Carreiras (2015) [5] are even more direct: *“the inconsistency of behavioral findings cannot and will not be settled by structural or functional brain differences”.* The reason, as they simply express it, is that “*there is no direct mapping between brain structure and cognitive function*” that enables one to link structural changes in a region or network to changes in cognition. 

Another critical assumption underlying a causal explanation for why bilingualisms should enhance EF is that language control is subsidiary to general cognitive control. Thus, it makes sense to look for that overlap between these two abilities in bilingual brains and, indeed, the evidence appears solid for a distributed frontoparietal network [6] involved in both types of control. However, Garcia-Pentón et al. [4] caution that: “*[…] demonstrating that both language and cognitive control mechanisms overlap does not necessarily imply a bilingual advantage. Thus, showing more than neuroanatomical differences between bilinguals and monolinguals is needed to underpin any possible bilingual advantage*”. Turning to the neuroanatomical evidence reviewed by Garcia-Pentón et al., there are no studies comparing the neuroanatomy of dual-language bilinguals to single-language bilinguals or to dense-code switchers and, consequently, we need to focus on the grosser distinction between bilinguals in general and monolinguals. Garcia-Pentón et al. [4] systematically reviewed 17 studies of the neuroanatomy of bilingualism. Their relevant conclusion was that the experimental evidence for structural changes in the brain due to bilingualism is “*relatively weak*” in that consistent and reproducible structural changes tend to occur in only one region, namely, the IFG. Furthermore, the differences that are found are often shrouded in the fog of valence ambiguity. For example, in the nine cross-sectional studies of white matter several showed differences in either the corpus collosum or inferior fronto-occipital fasciculus, but four showed increases in fractional anisotropy while three showed decreases. Garcia-Pentón et al. attribute the inconsistency in the findings to the heterogeneity of both the methods used and the types of bilinguals. Regarding the latter, proponents of the ACH could justifiably hypothesize that systematic differences in the predicted regions would emerge, if large samples of dual-language bilinguals are compared to those dominated by single-language experiences and/or to monolinguals. However, that work remains to be done. If different brain structures do not align with differences in performance on tasks requiring EF, then it could be because bilingualism induces changes in structure that serve other (non-executive) functions that neither improve nor impair general cognition [7]. This is compatible with the controlled-dose hypothesis that Paap [8] proposed. The gist of this hypothesis is that the early stages of L2 acquisition require domain-general EF, but that as fluency is gained and ubiquitous practice accrues, bilingual-language control (like any other complex skill) becomes increasingly automated with new mechanisms intimately tied to the specific domain. This argument is further discussed by Paap et al. [2] in their paper titled *On the encapsulation of bilingual language control*.

This fits the evidence reviewed by Garcia-Pentón et al. regarding the effects of age-of-acquisition (AoA) of L2 and L2 proficiency on the brain. They observed that the most consistent finding is in grey matter within the IFG and the white matter connecting the IFG with other regions. The ACH predicts that the degree of involvement or activation changes as a function of L2 proficiency such that there is less involvement as L2 is mastered and automatized. This perspective attributes the decrease in activation to a more efficient general control process that is associated with greater GM volumes and WM connectivity. However, instead of generating a more efficient “general” control process, it could reflect a shift from domain general processing to language specific processing encapsulated and specialized within a language processing system. 

There is an intriguing parallel between Paap’s controlled-dose hypothesis [8] and Pliatsikas’ Dynamic Restructuring Model (DRM) [9], although the former is a cognitive–functional model based on behavioral performance and the latter is neuroanatomical based on structural adaptations. Both models trace the acquisition of skilled bilingualism from initial exposure to peak fluency and emphasize that peak efficiency is achieved by acquiring new structures specific to the task. The DRM is a three-stage model that assumes that structural adaptations are dynamic and depend on the quantity and quality of the language learning and switching experience.

In the early stages, the DRM assumes that a restructuring occurs even when bilingual control is continuously practiced as would be the case during an extended period of L2 immersion. This process is referred to as the expansion–partial renormalization hypothesis (EPH). According to the EPH, learning of a skill leads to local generation of new dendritic spines in the region that undertakes the skill learning, which in turn provide an increased number of neural pathways compared to pre-training. This allows the most efficient circuits to be consolidated. Thus, an initial increase of local tissue is followed by a decrease due to pruning. Thus, once the most efficient networks have been identified and are in continuous use, both pre-training spines and underutilized post-training spines are eliminated. Pliatsikas reviewed many studies showing that training in a non-native language leads to increases in the volume of grey matter regions including but not limited to, regions related to language learning and use in the left hemisphere and a cluster of prefrontal regions related to switching between and controlling the production of the available languages in bilinguals. Since decreased diffusivity in the white matter is thought to signify more efficient communication between brain regions, it appears that second-language learning ‘forces’ the entire system to reorganize to accommodate the task of controlling for the selection of lexical, semantic, and phonological alternatives during production. Notably, the few studies that retested their participants several months after the completion of the training course reported a reduction of the initially observed restructuring in both grey and white matter. However, in contrast to the EPH, Pliatsikas [9] allows it might be the case that for white matter continuous exposure to a non-native language is a prerequisite for the ‘enhancement’ of structural connectivity. For the present purposes, the key conclusion is that *“…evidence from training studies clearly demonstrates that additional language learning and control is a form of skill acquisition that result in structural changes in a similar way that other skills do….”,* p. 461. 

In an optimistic closing, Pliatsikas suggested that the DRM may be able to explain the variability in the literature, because bilingualism is a dynamic experience that causes continuous adaptations in brain structure, which themselves depend on the language learning and switching demands of the linguistic environment and the amount of prior experience in meeting those demands. However, he is also fair in noting that “*The DRM aspires to be the first attempt to integrate and reconcile all the seemingly contradictory findings in the literature on bilingualism-induced structural neuroplasticity*”, p. 467. Given that the effects of intensity, use, and timing of bilingualism on neural structures are nonlinear, it is unlikely that differences in structure can accurately predict differences in bilingual language control, much less, differences in far transfer to tasks measuring domain-general EF.

### 2.2. Event-Related Potentials (ERPs)

ERPs, given their high temporal resolution, potentially provide strong evidence for testing whether the neural correlates of executive control processes are differentially strengthened by different types of bilingualism. Furthermore, behavioral measures represent the final output of interactions among several cognitive processes in the performance of a given task. Thus, ERPs are potentially more sensitive than behavioral measures because they allow us to detect modulations of a specific correlate of a single process along the chain of cognitive processing. 

Before focusing on the narrower research question of evidence for or against the ACH, it is worthwhile to ask if ERPs support the general hypothesis that EF is strengthened by bilingualism. In a recent systematic review, Cespón and Carreiras [10] concluded that the existence of a bilingual advantage in neural processing related to EFs remains uncertain and further studies are required. Resonating to critical challenges that we have discussed repeatedly [11,12,13] Cespón and Carreiras [10] argue that ERP measures often suffer from an alignment problem, valence ambiguity, and kind ambiguity. The alignment problem occurs when the pattern of behavioral differences across language groups is different from the pattern of neural differences (see [12,13] for several examples). Cespón and Carreiras [10] recognize the alignment problem in stating that “*modulations of neural activity in the absence of behavioral differences may lead to ambiguous conclusions*”, p. 320.

Valence ambiguity occurs when larger amplitudes are inconsistently and opportunistically interpreted as reflecting better or worse EF. Kind ambiguity occurs when a specific neural marker or locus is associated with multiple types of cognitive processing. Moreover, given the correlational nature of these studies, it is not possible to strictly establish the existence of a cause–effect relationship between bilingualism and enhanced EF. Central to the predictions derived by Cespón and Carreiras (2020) is the view that a valid conclusion for a bilingual advantage in an ERP marker requires establishing a correlation between that marker and behavioral performance. 

Although ERPs may reflect differences in neural processing that are not strong enough to produce behavioral differences, Cespón and Carreiras [10] recommend caution that only specific types of differences (not just any difference) in ERP latencies or amplitudes provides convincing support for a bilingual advantage. In deference to resolving valence ambiguity, they urge that a specific ERP signature needs to be closely related to the increased effort required on highly demanding trials of EF tasks such as the incongruent trials of Stroop, Simon, or flanker tasks. If differences in latency or amplitude can be tied to increases in effort, then it becomes clear that a difference aligning with less effort reflects better efficiency and a bilingual advantage in EF. Applying these principles, Cespón and Carreiras generated 18 specific ERP outcomes that would support a bilingual advantage in EF (see their Figure 1). To provide a concrete example, we revisited the magnitude of the N2 amplitude that was the focus of [13]. We argued that, based on previous published research, smaller N2 amplitude are associated with better EF and this matches the valance interpretation generated by Cespón and Carreiras. In their systematic review (see their Table 3), all 15 relevant studies reported zero N2 amplitude-differences that were in the *advantage* direction even though 4 of the 15 showed behavioral advantages. Across all 62 ERP tests, there were only six significant ERP *advantages* with three of those associated with the Go/No-Go task. In conclusion, the Cespón and Carreiras review provides very little evidence that ERP markers can consistently reveal bilingual advantages or that they are more sensitive than behavioral measures. Indeed, there were only two studies where a bilingual advantage was detected in an ERP marker when the behavioral tasks showed no advantage. From the standpoint of power, the disappointing results for ERP markers are not surprising as all 11 studies are starkly underpowered.

## 3. Far Transfer Predictions Derived from the Adaptive Control Hypothesis

The next major section reviews the spate of recent articles that test the far-transfer predictions of the ACH. This piqued the interest of our lab, because the eighth most-cited article in the *Journal of Cognitive Psychology* (with 207 google-scholar citations) is our article *Are bilingual advantages dependent upon specific tasks or specific bilingual experiences?* [14], to which we answered “no” based on the evidence at that time. The first set of five studies focus on the most straightforward and clear prediction, namely, that DLC experiences should lead to enhanced inhibitory control and switching ability. In each study, the procedures used to measure the three interactional contexts are carefully described, because one important purpose is to evaluate the strengths and weaknesses of these measures and provide recommendation for future research. The second set of studies focused on the predictions driven by DCS and the original ACH assumption that language mixing within utterances is conducted in an open control mode that should not enhance inhibitory control or switching ability. This section asks the question: Are there no benefits to executive control from dense code-switching? This section discusses the cogent arguments raised by Hofweber and Treffers-Daller [15,16] that DCS should not be operationally defined as switching within utterances, that only “congruent lexicalizations” should be considered as true DCS, and that true DCS is sometimes associated with better inhibitory control. A discussion of Green and Wei’s [17] updated ACH, rebranded as the Control Process Model (CPM), resolves some of these issues. A third major section considers the changes in neuroanatomy and various components of ERPs as an L2 is initially acquired and then as high levels of proficiency are obtained through extended immersion or training. All these sections set the stage for a large-scale reanalysis of data from two of our published studies [2,18] that is based on the strategy of identifying homogeneous and relatively pure groups of DLC, SLC, DCS bilinguals and monolinguals. By way of preview, there was never a significant difference between language groups across nine different measures of EF. 

### 3.1. Does DLC Lead to Better Executive Functioning?

Kalamala, Szewczyk, Chuderski, Senderecka, and Wodniecka (2020). The excellent study by Kalamala et al. [19] will be considered first, because four of the seven processes specified by Green and Abutalebi involve conflict resolution and the main strategy used in this study is to determine if a measure of the *intensity of DLC* predicts four measures of response inhibition. The Kalamala et al. study differs from those that partition participants into different groups and, for example, test whether a DLC group is better than an SLC group. Rather, a single *intensity of DLC* measure is computed for each of the 195 Polish–English bilinguals that can be used as a predictor of the inhibition factor in SEM or regression models. 

This strategy compels a composite measure that reflects not only the degree of language co-occurrence in the same interactional context, but also the degree to which the participant belongs in a different group, namely, DCS. Let us first examine language co-occurrence, which is the extent to which multiple languages are used in the same context. Similar to Hartanto and Yang [20] a questionnaire is used to probe the proportion of time that Polish and English are used at home, work, school, and free time. In the case of a bilingual, the purest DLC experience would be using both languages exactly half the time in each of the four contexts. The questionnaire solicits these proportions by asking: how many hours in a specific context (e.g., home) do you use Polish? Likewise, it is asked: how many hours do you use English? The proportions of using Polish and English (and any other languages) are combined for each context using Shannon’s entropy formula as shown in Equation (1).

(1) $$\sum_{i=1}^{n}-p_{i}log_{2}p_{i}$$

where *p_i_* is the probability of the use of a language in a context (see Kalamala et al. [19] for discussion). When languages are equally likely, entropy is high and when only a single language is used, it is zero. The final step in computing language co-occurrence for a given participant is to average the entropy scores across the four contexts while weighting them by the proportion of time spent in each context. Low entropy scores reflect mostly SLC experiences whereas high scores reflect mostly DLC experiences.

Intensity of DLC = language co-occurrence + language mixing within utterances(2)

Intensity of DLC = z(average entropy) + z(reverse(mixing within utterances))(3)

However, given that the ACH assumes that the open control mode used during dense code-switching obviates the need for goal maintenance, conflict monitoring, and inhibition, the entropy scores need to be adjusted for the amount of language switching that is within utterances/sentences. This adjustment, language mixing in Equation (1), is based on three items that highlight different types of dense-code switching:

(a). Word Insertions, {I use words in another language than the one I am currently speaking in, e.g., I say “My new teacher is *mila* (easy going)”}
- (b). Alternations, {I begin a sentence in one language and finish it in another, e.g., “Pożyczę Twój długopis, if you don’t mind”} and
- (c). Mixing within Words {I mix languages within one word, i.e., I blend a Polish word ending with an English word or vice versa, e.g., “spotkajmy się za cornerem”.

Each item was responded to with a 9-point Likert scale ranging from 1 NEVER to 9 ALWAYS. 

As shown in their Equation (2), the language mixing measure is the average of the three types after the ratings have been reverse scored. Reverse scoring these items means that participants who engage in very little mixing within utterances will have the largest scores while those who frequently engage in dense-code switching will have smaller mixing scores. Thus, the *intensity of DLC* measure for a participant who rarely switches within an utterance receives a bigger bump compared to dense-code switchers. Note that the operational definition of intensity of DLC treats the two components as equally important. 

A potential problem with this intensity of DLC measure is that it assumes that word insertions and alternations are the same as congruent lexicalizations, but in an extension of the original ACH, Green and Wei [17] assume that insertions and alternations require coupled control that could contribute to an enhancement of general inhibitory control. Thus, Kalamala et al.’s formula may over correct for the type of dense-code switching that reduces the effectiveness of general DLC. 

Setting the potential problem of the measure aside, the results are unambiguous. Each participant completed four tasks that in this study cohered into a latent variable plausibly labeled *response inhibition*: antisaccade, Stroop, go/no-go, and stop-signal. *Intensity of DLC* did not predict response inhibition in a series of SEM models, nor did it predict performance in any of the analyses of individual tasks. The original ACH assumed that using different languages in the same situation without mixing them in single utterances (i.e., DLC bilingualism) engages and consequently trains response inhibition in bilinguals. Although the study provided highly reliable measures of bilingualism and inhibition, the results did not support the predictions derived from the ACH. The results suggest that bilinguals who operate in a DLC context either do not engage a domain general mechanism of response inhibition to control language production or engage it to the same extent as other bilinguals. This led Kalamala et al. to: “*…conclude that the ACH probably does not account for the discrepant results of studies testing the relationship between bilingualism and cognitive control efficiency, at least with respect to response inhibition*”, p. 12. One might attempt to deflect these null results by pointing out that the ACH emphasizes that language selection initially takes place at the conceptual level and, consequently, that far-transfer effects should be visible for interference suppression, not response inhibition. However, Green and Abutalebi [1] specifically noted that *“….adaptive effects should be evident in the analysis of reaction time distribution for conflict tasks such as the color-word Stroop tasks*”, p. 522 (one of the four tasks comprising the latent-variable in Kalamala et al.) and even more specifically state that *“Our analysis leads to the prediction that bilingual speakers in the dual-language context will be the more proficient in inhibition than those in the single-language or dense code-switching contexts”,* p. 522. 

Hartanto and Yang (2016). The study by Kalamala et al. has its roots in the seminal article by Hartanto and Yang [20]. Hartanto and Yang were the first to show that bilinguals with DLC tendencies (n = 75) had better executive control (in the form of smaller switching costs) compared to those with SLC patterns (n = 58).

Using a 5-point Likert scale (1 = never, 5 = always), participants reported on two items regarding the extent to which they used two languages within the same context (DLC bilingualism) and in different context (SLC bilingualism, reverse coded). Scores on these two items were summed to produce a composite score for DLC. The first item (directly tapping DLC) was *“Do you speak two or more languages interchangeably within the same situation in general (e.g., using both English and Chinese at school)?* The second item (directly tapping SLC) was “*Do you speak only one language in one environment in general (e.g., using Chinese at home but English at school)?*” Hartanto and Yang (2016) explicitly assumed that DLC bilingualism is the polar opposite of SLC bilinguals, both of which fall along a bipolar continuum. Thus, both items are assumed to be measuring the same construct and a composite can be formed once the SLC item is reverse coded. This yields a DLC scale of 2 to 10 with greater scores signifying more DLC bilingualism. A mean split was used to partition the participants into DLC and SLC bilinguals. 

Executive functioning (EF) measures were derived from a color–shape switching task in the form of switching costs (switch trials minus repeat trials) and mixing costs (repeat trials in the mixed block minus pure task). Analyses of both speed and accuracy showed that the only significant group difference was that the DLC group had smaller RT switching costs than the SLC group. The result for switch-cost RT was consistent with the predictions of the ACH, whereas the null result for mixing-cost RT was not.

A set of regression analyses on the switch costs are more directly comparable to the analyses in Kalamala et al. In all models, paternal education, verbal ability, and nonverbal intelligence were entered in Step 1. Model 2 includes an *Index of SLC* that operationally is the discrepancy between the percentage of time L1 was used and the total for all other languages aggregated across the four contexts. If SLC and DLC are polar opposites then the *Index of SLC* should also predict switch costs just as the composite DLC score did in Model 1. However, the overall Model 2 was not significant and, of course, neither was the *β* Index of the *SLC* predictor. 

In summary, some of the Hartanto and Yang (2016) results support the possibility that DLC tendencies enhance switching ability (reduce switch costs) while the Kalamala et al. results showed no relationship between DLC proclivities and response inhibition. Of course, there are major differences between the studies. Perhaps DLC tendencies enhance switching ability, but not response inhibition or, the validity of the measures of DLC experience might differ. Kalamala et al. questioned the validity of Hartanto and Yang’s composite measure of DLC bilingualism and here is their logic. Recall that Hartanto and Yang’s measure of DLC bilingualism is a composite based on a DLC probe and a reverse coded SLC probe:

Item (1): “Do you speak two or more languages interchangeably within the same situation in general (e.g., using both English and Chinese at school)?” 

Item (2): “Do you speak only one language in one environment in general (e.g., using Chinese at home but English at school)?” In their Appendix B, Kalamala et al. report that the correlations between these two items are rho = −0.25 in Hartanto and Yang’s (2016) original study and rho = −0.36 in their replication. Because the N’s are large (133 and 195, respectively) the correlations are significant at *p* < 0.001, but the magnitudes suggest that no more than 10% of the variability in Item 2 scores can be predicted from the Item 1 scores. This suggests that a strong assumption that these two items tap opposite ends of the same continuum is suspect. To summarize, Hartanto and Yang have two measures of the same construct (extent of DLC experience): the composite score of DLC based on Items (1) and (2) above and *the Index of SLC* based on proportion of use across contexts. The former predicted switching cost, but the latter did not. Kalamala et al. argue that the *Index of SLC* is more valid than the DLC measure. If the SLC measure is given greater weight, then both studies converge on the conclusion that DLC tendencies do not predict executive control.

Hartanto and Yang (2020). Four years later, Hartanto and Yang (2020) [21] agreed that DLC and SLC should not be measured as two ends of a continuum. They shifted their strategy to directly asking bilingual participants to estimate the degree to which they engage in SLC, DLC, and DCS in each of the four contexts (home, school, work, and free time). The critical items are:

SLC Item—*I speak only one language and rarely switch to the other language at home*.

DLC Item—*I speak two (or more languages) when I converse with different speakers at school. I often switch languages but rarely mix languages within an utterance*.

DCS Item—*I routinely mix two (or more) languages within an utterance to most speakers at work*.

A composite for each of the three tendencies is calculated by weighting the responses to each context by the percentage of time spent in the specific context. In contrast to Kalamala et al., the instructions for percentage of time at home are not adjusted for time asleep. This is the first article reviewed to this point that uses probes that clearly distinguish between the three modes. If bilinguals can accurately estimate these relative frequencies, then this is a very direct way of measuring the three tendencies described in the ACH.

Putting aside concerns about the validity of these measures until our final discussion, the Hartanto and Yang (2020) study provides a strong test of the hypothesis that DLC experiences should enhance EF compared to SLC proclivities. These three indices were computed for each of the 175 bilinguals. Three different task measures were used to identify latent variables for inhibitory control, switching costs, mixing costs, and working memory. The measures of DLC and DCS were included in the SEMs with the index of SLC as the reference group. The first important result is that DLC did not significantly predict the latent variable for inhibitory control that was based on three versions of the flanker task. This is consistent with the results of Kalamala et al. (2020) with the caveat that the flanker effect is often thought of as *interference control* while Kalamala’s latent variable was assumed to reflect *response inhibition*. Given that both studies employed a large sample size and used latent-variables to measure EF, these results do not provide evidence for the ACH prediction that DLC experiences, compared to SLC experiences, lead to advantages in inhibitory control. 

In contrast to the *inhibition* results, all of the SEMS showed that the *index of DLC* significantly predicted the latent variable of switching costs. This replicates their earlier result (Hartanto and Yang, 2016) with a different measure of DLC bilingualism. The newer study also included latent variables for *working memory* and *goal maintenance* (mixing costs) and DLC did not significantly predict either of these components of EF.

Lai and O’Brien (2020). Lai and O’Brien [22] conducted the most ambitious and direct test of the ACH. Starting with the general predictions formulated in Table 1 of Green and Abutalebi that specify which control processes are demanded in each of the three interactional contexts, Lai and O’Brien make specific predictions (shown in their Table 1) for the five processes assumed to be engaged in DLC: (1) goal maintenance, (2) conflict monitoring, (3) interference suppression, (4) selective response inhibition, and (5) task engagement and disengagement. The specific predictions are operationally defined in terms of measures derived from a verbal (Stroop) and a non-verbal (global–local) interference task that also involve cued switching between the dimensions. For example, engagement and disengagement is defined as the RT difference between switch and repeat trials in the mixed block. The two types of tasks and five measures of control provide a total of ten dependent variables. 

A first set of analyses determined if a DLC intensity measure derived for each of 74 Mandarin–English bilinguals predicted EF. This measure of DLC intensity is based on combinations of responses to these six items:

1. I tend to speak only one language in one environment and another language in another environment (SLC),
2. I tend to speak both languages in the same environment (DLC) Note that this item is another example of an item that cannot be taken as a pure indicator (of DLC in this case) because it also includes DCS
3. I switch languages between sentences when conversing with others (inter-sentential),
4. I tend to switch languages during a conversation (general switching),
5. I include Chinese words or phrases into the English conversations I have with others (intra-sentential), and
6. I include English words or phrases into the Chinese conversations I have with others (intra-sentential).

The DLC intensity measure was the sum of responses to (2) and (3). A series of 10 hierarchical regressions were conducted for each of the 10 EF measures as the outcome variable. Age and a measure of balanced bilingual proficiency were entered in the first and second step, respectively, and DLC intensity in the third step to see if DLC bilingualism could explain EF over and above bilingual proficiency alone. For the switch costs derived from the Stroop task, the results were ambiguous as the *β* coefficient for DLC intensity was −0.22, *p* = 0.06. The results for switch costs in the global–local task were unambiguously null. It may be fair to say that the Lai and O’Brien results weakly support the DLC advantages in switch costs reported by Hartanto and Yang [20,21]. Lai and O’Brien speculated that their sample of Mandarin–English bilinguals living in Singapore may not sufficiently differentiate themselves to offer a strong test of the ACH because of the pervasive multilingualism in Singapore. The means and SDs were 8.67 (2.60) for engagement in SLC and 6.03 (1.90) for DLC. Of course, Hartanto and Yang were also testing multilinguals from Singapore and may also have been challenged by a restricted range, but that did not prevent them from observing significant differences in switch costs. 

To revisit the point made by Kalamala et al., items (1) and (2) are intended to discriminate SLC from DLC but, as usual, the correlation between the two is significant, but small (r = −0.28, *p* < 0.05). Note also that items (5) and (6) that are intended to capture language switching within sentences do not explicitly state that the participant should only consider intra-sentential switches. Given the possibility that some participants did not consider whether switches are within or between utterances, it is not surprising that the correlation between (5) and the item that specifically refers to switching between sentences (3) is *r* = +0.58, *p* < 0.01. It seems plausible that the lack of evidence favoring the ACH in this analysis and other analyses based on self-reports is that the items are interpreted differently across participants and that participants are not highly accurate in interrogating their episodic memories while selecting a response on the Likert scale.

Another aspect of Lai and O’Brien’s study—a wonderfully ambitious study—was the inclusion of various objective measures of language switching: (1) a verbal fluency task where participants had to alternate languages, (2) the frequency of different types of switching produced in telling a familiar story with picture prompts and the instruction to use both Mandarin and English, and (3) the frequency of different types of switching produced in conversation with the experimenter about favorite childhood stories. A valuable, but disconcerting, finding was that all 15 correlations between the three self-report measures (SLC, DLC, DCS) and the 5 objective measures were non-significant. The correlation between tendency to engage in dense code-switching (based on items 5 and 6) and intra-sentential switches in a natural conversation was zero. However, hierarchical regression models using the objective measures of language switching did reveal two significant predictors of EF performance. The relative frequency of inter-sentential switching in the natural conversation task predicted goal maintenance in the global–local task, *β* = −0.31, *p* = 0.01. Likewise, it predicted conflict monitoring in the global–local task, *β* = −0.29, *p* = 0.02. However, these two significant outcomes are out of 30 regression models formed by the combination of three objective measures (alternating verbal fluency, story narration, natural conversation), two tasks (Stroop and global/local), and five measures of EF. Given the universe of tests in the Lai and O’Brien study, the relative number of confirmed predictions strikes us as unimpressive. However, if one highly values the ecological validity of the measures derived from the natural conversation, then one should not minimize that there is some evidence that inter-sentential switching predicted goal maintenance and conflict monitoring.

Pot, Keijzer, and de Bot (2018). Pot, Keijzer, and de Bot [23] tested for positive effects of DLC tendencies in a large sample (N = 387) of elderly (M = 72 years) multilinguals living in the northern Netherlands who typically spoke at least four languages. Data from an arrow version of the flanker task and the Wisconsin Card Sorting Task (WCST) were analyzed using linear mixed effects regression that included a large battery of lifestyle, health, wellbeing, personality, and language-use variables. The strengths of the study include the large sample size, the focus on older adults, the rich multilingualism in the sampled region, and the wide array of measures solicited from each participant. Although the simple number of languages used did not predict performance on the EF tasks, Pot et al. concluded that “*when different languages are used frequently in different contexts, enhanced attentional control is observed”,* p. 1. This sounds like support for the prediction that DLC bilingualism enhances EF. The basis for that conclusion is considered next.

For each language the degree of usage (5-point scale ranging from never to always) is assessed in each of four social domains: family, friends, neighbors, and acquaintances. The *intensity of language use* measure was computed for each language by taking the average score across the four contexts. Thus, a maximum score of 5 indicated that a specific language is always used across all four domains. A minimum score of 1 means that this language is not currently used in any of the domains.

The results for the linear mixed effects regression analysis of the flanker task are very complicated. First, there are three intensity of language-use measures, one for each of the three most dominant languages and only the L2 factor significantly predicted performance on the flanker task. Surprisingly, the direction signaled a DCL disadvantage, not an advantage. Second, the intensity of L2 measure interacts with the *contextual switching factor* derived from the BLSQ [24]. The three relevant questions from the BLSQ factor are:

Q1: *There are situations in which I always switch between the two languages,*

Q2: *There are certain topics or issues for which I normally switch between the two languages,*

Q3: *I tend to switch languages during a conversation.*

Q1, in particular, appears to be tapping into the same construct as the *intensity of language use measures* (that ask bilinguals to indicate the extent to which a language is used across four contexts). A nuanced perspective might point to the fact that probes for interactional contexts usually refer to locations (e.g., home, school, work) or people (e.g., family, friends, neighbors) whereas the BLSQ frames the probes in terms of topics (Q2) and explicitly refers to language switching. However, note that high agreement to the BLSQ questions should be correlated with DLC tendencies. Despite the appreciation that *L2 intensity of language use* and *contextual switching* appear to be measuring, at least in part, the same thing their interaction is significant. Bilinguals with high scores on both measures have significantly smaller flanker effects. A negative main effect of L2 intensity of language use that reverses at high levels of contextual switching seems unintuitive, but the authors arrive at the following conclusion. “*Without this interaction, the use of the second language across domains yields higher Flanker effect scores, suggesting that there needs to be some element of control/monitoring of attention to language cues that is present in this dual-language mode and which carries over into more general cognitive processes*”, p. 21. With respect to the other component of EF, the flexibility and/or inhibition recruited during the WCST, there were no significant effects of DLC bilingualism. 

The *intensity of language use* measure deserves closer examination. It is not a proper member of the family of entropy-like measures because separate measures are calculated for each of the three languages, and each is treated is a separate factor in the regression analyses. The entropy measure developed by Gullifer and Titone introduced above in Equation (1) describes a monotonically increasing function. As the overall proportion of use across the languages of a multilingual becomes more and more equal, the entropy scores monotonically increase. However, this study does not compute a composite across all languages. Thus, as the L2 *intensity of language use* increases its association with DLC tendencies is non-monotonic. Consider some concrete examples. An individual who never uses L2 in any of the four contexts has lower entropy (as calculated by Shannon’s formula) than someone who sometimes uses L2 in 50% of the contexts. However, as the dominance of any specific language grows, it steals intensity from its mates and will lead to lower entropy. The extreme case is easy to see. If someone uses L2 100% of the time across all four contexts, then the use of L1 and L3 must be 0% and entropy is at a minimum. Given this analysis of the *intensity of language use* measure, it is less surprising that it interacts with the *contextual switching factor* derived from the BLSQ. Another reason to interpret that interaction with extreme caution is that the model with the interaction performs only “slightly” better than the model without the interaction. In their conclusion, they suggest: “*It is especially this dual-language context, rather than a dense code-switching context, that incurs benefit in our sample*”, p. 21. This may be true, but as shown above the direction of any effect involving their *intensity of language use* measure for L2 is ambiguous and they did not measure DCS. Although it seems likely that the sample includes regions that support prevalent DCS, any role of DCS is this study is speculative.

### 3.2. Tentative Answer to Question Does DLC Lead to Better Executive Control?

Taking a conservative (or perhaps skeptical) perspective on the large, ambitious, and creative studies reviewed above, the evidence supporting the ACH prediction that DLC tendencies enhance inhibition appears to be inconsistent and overall weak. When inhibition is measured using a latent variable (Hartanto and Yang [21], Kalamala et al. [19]) the effect of DLC bilingualism is null or limited to a complicated interaction that is difficult to interpret (Pot et al., [23]). In contrast, Hartanto and Yang [20,21] reported DLC advantages in cued switching tasks that do cohere into a latent variable. The inconsistent evidence with respect to switching or mental flexibility includes Pot et al.’s finding that DLC tendencies did not predict performance on the WCST and Lai and O’Brien’s nonsignificant findings for switch costs in both the Stroop and global/local tasks. In summary, although other types of predictions derived from the original or revised ACH have yet to be considered, the principle prediction that DLC bilingualism has better domain-general EF in comparison to SLC bilingualism is limited to nonverbal switching and that evidence is inconsistent across studies.

### 3.3. Are There No Benefits to Executive Control from Dense Code-Switching?

The original ACH [1] assumes that DCS occurs in an open-control mode that consequently does not provide experiences that would enhance general EF. DCS is characterized as frequent switches within utterances or sentences. One strategy for testing this hypothesis is to compare bilinguals who engage in intense DCS to those who do not and might otherwise be considered SLC or DLC bilinguals. According to the ACH framework, DCS bilinguals should clearly perform worse than DLC bilinguals (who presumably exercise frequent inhibitory control) and perhaps less well than SLC bilinguals (who presumably exercise some amount of inhibitory control). 

### 3.4. The Effects of Switching within Sentences/Utterances

Hartanto and Yang (2016). The seminal Hartanto and Yang study [20] adopted the simple assumption that a good index of DCS and the open control mode was the frequency of intra-sentential language mixing. A set of regression analyses used switching costs as the outcome variable. In all models, paternal education, verbal ability, and nonverbal intelligence were entered in Step 1. All models included the *index of intrasentential code-switching* as one of the additional predictors in Step 2. An example of one item forming the index is: “*How often do you mix words of different languages when speaking at home (e.g., when you have trouble finding a word in Chinese, you tend to immediately replace it with an English word instead or vice versa)?*” Note that the item and the example focus the participants attention on mixing that involves word insertion rather than alternating at phrase boundaries or congruent lexicalizations. Across models the *β* for the index of intrasentential code-switching was positive (indicating it exacerbated switch costs), but was never significant at *p* < 0.05. Thus, there is a hint that frequent intrasentential switching has a net negative effect on switch costs, but it would be prudent to remember that the *β* was not significant despite the large sample size and desirable power. 

Although an index of intrasentential code switching was computed for each bilingual and used as a predictor in the regression models, it was never used to identify a group of super dense-code switchers. A similar strategy was employed in the Hartanto and Yang (2020) follow-up study where separate predictors were derived for SLC, DLC, and DCS tendencies. To refresh, one DCS Item was “*I routinely mix two (or more) languages within an utterance to most speakers at work.*” In the SEM analyses, higher exposure to dense code switching compared to the DLC was not associated with significant differences in either inhibitory control or switching costs.

Paap et al. (2019). Likewise, we [2] reported several analyses predicting composite interference scores from four tasks (Simon, vertical spatial Stroop, horizontal spatial Stroop, flanker) for 104 bilinguals who rated their P2 proficiencies in the range of 4 to 7 (see Table 2 for the full scale). As shown in Paap et al.’s Table 7b, frequency of switching within a sentence (as measured on a single 5-point Likert scale) did not predict the composite inhibition measure in either zero-order (*r* = +0.08) correlations or regression analyses that included Raven’s scores and an SES measure as control variables (*β* = +0.08). It should be noted that Paap et al.’s measure of mixing within sentences highlighted word insertions, “*When producing a sentence in one language bilinguals sometimes replace a word or two with its translation equivalent in their other language. How often do you use a word from another language you know?*” The same item was also included in an earlier study [18] and again, this measure of switching within sentences did not significantly correlate with any of the measures of EF: switching or mixing costs in a cued switching task, positive or negative slopes in a conjunctive visual search task, interference scores in a spatial Stroop task, or performance in a morphing ambiguous figures task.

Samuel et al. (2018). Samuel and colleagues [25], in a fascinating study that looked at the contribution of both culture and bilingualism on the Simon effect, had participants rate how often they used more than one language within one sentence on a 0–10 scale from “never” to “always”. Overall, their results offered no support for a bilingual advantage, but they did report evidence for an East Asian (Korean) advantage over a Western (British) culture. Most relevant to the present purpose, their frequency of switching within sentences measure did not significantly predict Simon effects in RT, but did predict larger Simon effects in accuracy in one of their three groups. 

Kang and Lust (2019). One substantial strength of the Kang and Lust study [26] is that they developed and used objective measures of inter- and intrasentential language switching in an investigation of 43 8-year-old English-Chinese bilinguals living in Singapore. Based on multiple measures, the children were highly proficient in both languages, although somewhat English dominant. In a task intended to induce inter-sentential language switching, the child was asked questions and instructed to answer as fast as possible and to say as much as they could in 20 s. Inter-sentential switching was primed by alternating between English and Chinese across the eight questions. In a second task, intra-sentential code switching was primed by simply including cross-language insertions in each of the eight questions. A measure of code switching (insertion frequency) was defined by the number of times a child inserted words, phrases, or other constituents from the other language in a sentence. The measure of EF was a “switch cost” measure derived from a semantic fluency task (name as many animals as you can in 30 s) that included single-language and mixed-languages blocks. In the latter, the child had to alternate responses between English and Chinese. Switch costs were defined as the difference in the number of correct responses in the mixed-blocks compared to the single-language blocks. Although those children who code-switched more during the conversation that modeled intrasentential switches incurred lower switch costs on the semantic fluency task, the relationship was not significant. This null result is consistent with the hypothesis that DCS occurs in an open-control mode that does not recruit domain-general EF and, consequently, does not enhance EF. However, this null correlation might reflect nothing more than a restricted range of code switching as all the children were highly proficient and engaged in code switching both by observer reports and their performance in the laboratory conversation. If the same experiment was conducted with a diverse sample of monolinguals and bilinguals and the results showed that EF performance first increased and then leveled off (or even decreased) as the frequency of intra-sentential switching increased, this pattern would strongly support the view that frequent inter-sentential switching across a variety of contexts induces advantages, but that DCS does not.

### 3.5. Different Types of Language Mixing within Sentences/Utterances

However, the simple prediction that code switching within utterances reflects an open-control mode that will not enhance general EF is in even greater jeopardy, because Hofweber and colleagues [15] reported that dense code-switching sometimes yields better EF compared to either SLC or DLC proclivities. This formidable challenge to the hypothesis that dense code-switching uses an open control model that eschews the need for inhibitory control starts with a theoretical challenge arguing that dense code-switching should not be equated with all forms of switching within an utterance. Rather, following Muysken [27], one should consider three types of switches that can occur within a single sentence: (1) alternation, that is, switching at a phrase boundary, (2) insertion of a word from the non-matrix language, and (3) congruent lexicalization, i.e., a type of code-switching in which no clear line can be drawn between the involved languages. All bilinguals engage in the first two to some extent and when they do, Hofweber et al. assume that they recruit top–down global inhibition. In contrast, congruent lexicalization (i.e., true dense code-switching) predominantly occurs in established bilingual communities with several generations of language contact. Hofweber et al. assume that congruent lexicalization occurs in the absence of global inhibition, but that it relies heavily on monitoring for conflict between the languages and resolving the competition through local inhibition. This leads to the prediction that bilinguals who frequently mix languages through congruent lexicalization may have better inhibitory control (as reflected in smaller flanker effects), especially when the proportion of congruent and incongruent trials are equal and place a premium on monitoring each trial for possible conflict. Indeed, the mean flanker effect for 11 5th generation German–English bilinguals living in South Africa (M = 48 ms) was significantly smaller than the mean (80 ms) for 11 1st generation German–English bilinguals living in England. 

The 5th generation German–English bilinguals living in South Africa fit the situation where congruent lexicalizations (true DCS) are assumed to evolve and thrive. This assumption was put to the test using a frequency judgment task. In this task, bilinguals were presented with 56 “real” utterances containing 14 code-switches of each type counting the insertion of German into an English phrase and the mirror image as two separate types. Participants were instructed to imagine that they were having an informal conversation with a German–English bilingual friend and were asked to rate the frequency with which they would encounter utterances like the example provided on a scale from “1” = never to “7” = “all the time”. Although we are not aware of any direct comparisons between this frequency judgment task and probes that simply ask bilinguals how often they, for example, insert a German word in an English phrase, the intuition is that the frequency judgment task may enjoy both better validity and reliability. In any event, the mean for dense code-switching was significantly higher in the 5th generation group from South Africa compared to the 1st generation group living in England.

As alluded to above, another important aspect of the Hofweber et al. design is that they manipulated expectancy in their flanker task across blocks. The need for monitoring for conflict was assumed to increase as the proportion of congruent trials decreased from 92% to 75% to 50%. Furthermore, they assumed that constant monitoring is required in true dense-code switching and, consequently, it is the 50–50 condition that should yield smaller conflict effects for the 5th generation (high DCS bilinguals) compared to the 1st generation (low DCS bilinguals). As shown in their Figure 5, this is exactly the pattern of interaction they obtained.

In contrast to all of the other studies reviewed so far, the sample size is very small and that increases the risk of both Type 1 and Type 2 errors [28]. Fortunately, this group has succeeded in a conceptual replication that compares Turkey–English bilinguals born in Turkey (n = 39) to those born in Cyprus (n = 28). Those born in Cyprus were predicted to use more dense code-switching compared to the Turkish group, but the frequency judgment task did not fully support the prediction (*p* = 0.063). Nonetheless, the Cyprus born group showed a significantly smaller (*p* < 0.001) flanker effect than the Turkey born group and both groups of bilinguals had significantly smaller flanker effects compared to a group of British English monolinguals (n = 30). In summary, there is no evidence that frequent switching within utterances diminishes performance on tasks purported to measure EF as would be predicted by the simple hypothesis that an open control mode is the dominant mode for all types of switches within an utterance.

Green and Wei’s (2014) control process model. To be fair, Green and Wei [17] offered an update of the ACH that addresses some of the problems under discussion. The update is referred to as the *control process model* of code-switching. It embraces the typology of Muysken [27] and assumes that there are process differences across types. “*In the case of alternation, activation would shift from one language to another, and in the case of insertion, activation in one language would be temporarily diminished. For congruent lexicalization, the two languages partially share their processing systems*”, p. 500. Schema coordination either restricts the entry of items into the competitive queuing network to one language (when coordination is competitive) or allows items from either language (when coordination is cooperative). The control process model maintains the assumption that both SLC and DLC require a competitive relationship between the language schema where one schema dominates to the exclusion of the other. It also maintains the critical distinction that in the SLC words from the non-target languages are inhibited for the duration of the current context, but that in DLC control passes back and forth from one schema to another depending on the languages spoken by the addressee. Thus, the DLC requires more frequent doses of *competitive control*. Critically, the new control-process model adds a new distinction for switching within utterances. Cooperatively mixing languages within a sentence can occur in either a *coupled control* mode or an *open control* mode. An analogy to a “latch” is used to characterize coupled control—a highly activated word form from the non-target language can push open the latch, but it closes when this relevant item enters the next stage of processing. In the coupled control model, the language schemas play no top–down role in determining the output. In other words, mixing within utterances by word insertion does not require schema inhibition, because there is no disengagement of the first schema. 

Green and Wei [17] made some explicit predictions for their control process model. “*A dual language context should enhance cognitive control over that displayed in a single-language context because of the greater potential for interference and the need to control it. By contrast, a single-language context may require enhanced cognitive control relative to a dense code-switching context, because it is vital to avoid switching between languages in a single-language context*”, p. 506. Interestingly, they observed that if the same competitive control mode was universal then dense code-switching would be intensely demanding of cognitive control. Although they find this implausible in light of the fluency exhibited during dense coded-switching, it is exactly the position, prediction. and outcome of Hofweber and colleagues.

### 3.6. Does Language Mixing (a Transient Dual Language Context) Trigger Better General EF?

More transient effects of language mixing on interference control were first reported in a new paradigm introduced by Wu and Thierry [29]. Bilinguals performed a flanker task that concurrently presented task-irrelevant words in either a single language or two languages under the supposition that mixing words from two languages would activate both languages and the domain-general conflict resolution processes used to resolve the competition during the normal course of bilingual language control. Consistent with that prediction, the flanker effect (for accuracy) was smaller in the mixed-language condition. Furthermore, “*This result was supported by…. reduced amplitude of the P300, an electrophysiological correlate of cognitive interference*”. However, the review conducted by Cespón and Carreiras [10] led them to conclude that larger P300 amplitudes reflect “increased performance”. Thus, the reduced P300 amplitude observed does not reflect better performance. A similar reduction in P300 amplitude was reported by Jiao and colleagues [30] in a study discussed below. This could be another case where confirmation bias leads authors to interpret an “ambiguous” neuroscience outcome as a positive and supporting outcome [31].

In any event, Timmer, Wodniecka, and Costa [32] attempted a reasonably close replication of Wu and Thierry (2013) using Catalan–Spanish bilinguals and the ANT task. In this study, the P300 amplitude increased (reversing the results of Wu and Thierry) in the mixed-language condition, but there were no behavioral differences across conditions. There was clearly no evidence that a mixed language context triggered better inhibitory control, although Timmer et al. do assume based on the ERP signatures that mixing enhances the alerting network. They also revealed an extremely important new result in the last two sentences of their manuscript: “*…does switching between non-linguistic materials (e.g., low-level perceptual color categories like red and blue) also produce enhanced processing during alerting? We address this question in follow-up studies that revealed an absence of similar enhancing effect with non-linguistic switching and its presence when switching between linguistic categories (e.g., nouns and verbs)”,* p. 38. If these “mixing” effects can be triggered by within-language switching, this appears to seriously challenge interpretations based on the unique benefits associated with a lifetime of switching between two or more languages. 

Yang and colleagues [33] conducted a conceptual replication of Wu and Thierry [29] but with several substantial changes. The three language contexts were established in a first task (cued picture naming) rather than being completely incidental. In each context, trilinguals were cued on each trial to name the picture in one of two languages. The trilinguals were highly fluent in L1 (Cantonese) and L2 (Mandarin), but less fluent in L3 (English). The contexts differed only with respect to the specific languages paired during a picture-naming block: L1 and L2, L1 and L3, or L2 and L3. The prediction derived from the ACH was that the L1–L2 pairing should activate the inhibitory control network typically used during dual-language context and thereby prime the domain-general process used in the flanker task that immediately followed the picture naming. Another major difference is that fMRI activations in regions of interest were used rather than ERPs. 

The behavioral results and their interpretation are disconcerting. First, although the flanker effects (RT differences between congruent and incongruent trials) were significantly greater than zero and, in fact, typical in magnitude for young adults, there were no significant differences across the three contexts. For young adults, flanker effects in speed are typically the more sensitive measure compared to flanker effects in accuracy. However, the flanker effects for accuracy rates “*suggested a facilitation effect of the L1-L2 context on inhibitory control processes*”, p. 7. The basis for that interpretation was that in a series of t-tests the flanker accuracy effect was significant in the L1–L3 and L2–L3 contexts, but not in the L1–L2 context. Of course, this does provide direct statistical evidence that the L1–L2 context was associated with a smaller interference effect than the other two contexts. Their conclusion would be supported if the Trial Type (congruent, incongruent) × Context (L1–L2, L1–L3, L2–L3) interaction was significant. Inspection of their Figure 2 suggests that the interaction was not significant. Thus, contrary to the impression given by the authors’ narrative, there is no evidence that the differences in fMRI activation can be linked to better inhibitory control in the L1–L2 context. This alone undermines the impact of the study.

One might also note that making the dual-language context part of a task rather than being incidental opens any real effect of context to a plethora of alternative explanations. One such plausible alternative follows from noting that the Yang et al. design maps onto the typical depletion-task then outcome-task paradigm often used to test Baumeister’s [34] ego-depletion theory. If switching between two languages of high fluency (the L1–L2 context) is easier and hence less depleting than the contexts involving L3, then performance on the difficult (incongruent) trials of the flanker task should be easier compared to the two other contexts. Indeed, interference tasks such as the flanker are often used in the literature on ego depletion effects. 

Several additional studies have adopted this paradigm of alternating trials between a language task and a flanker task rather than manipulating language context in an incidental manner. Two studies by Jiao and colleagues [30,35] used a picture–word verification task where languages were mixed in one block, but always English or always Chinese in the pure blocks. These experiments consistently show global RT advantages in the mixed condition, but usually no advantage in the flanker interference effect. Jiao et al., [35] is the exception and only for the contrast between the mixed condition and the pure L1 block. Even though global RT is often used as a measure of the monitoring component of EF, it does of course reflect all the perceptual, cognitive, and motor processes operating in a specific task and, consequently, is a very impure measure of any one of them. Jiao and colleagues are aware of this and to their credit reported two control experiments in [35] that removed congruency from the “flanker” task by using only neutral flankers. Their results showed that the language effect on global RT only occurs when the task involves conflict resolution. This certainly helps, but the alerting advantage favored by Timmer et al. may be muted by ceiling effects when the standard (resource demanding) flanker task is replaced by the easier version where all trials have neutral flankers. All of this is intriguing, but additional research is needed to confirm the hypothesis that language mixing induces a transient boost in monitoring or inhibition. This is especially true, given Timmer et al.’s demonstration that switching linguistic categories within a single language produces the same enhancements as mixing languages.

Yet another provocative study interleaved reading a sentence with the flanker task [36]. When the sentence involved a language switch the flanker interference effect was significantly smaller compared to single-language sentences. If one thinks of code-switching as a process that requires conflict resolution, as the authors do, then the results are “*consistent with conflict adaptation*” as the flanker congruency effect is smaller when immediately preceded by an “incongruent” (code-switched) sentence. This result would be very surprising to those familiar with the conflict adaptation literature since it is exceeding difficult to generate congruency sequence effects (CSE)—the hallmark of conflict adaptation—when the tasks alternate [37]. Furthermore, when CSEs are derived from multiple tasks, the intertask correlations are near zero [38]. CSEs appear to be task specific [39]. If the Adler et al. results [36] are true conflict adaptation effects, they are unprecedented.

As a final footnote to the many studies that use the flanker task and concomitantly record neuroscience measures, the recommendation of Paap and colleagues [40] is to stop using the flanker task, because it shows no convergent validity with other tasks used to measure inhibitory control or attentional control. If the flanker interference task does not provide a domain-general measure of inhibition, then there is no reason to expect that ubiquitous practice in dual-language bilingualism would positively transfer to the flanker task. In fact, if interference control in the flanker is task specific any such associations with bilingualism constitute a puzzle. 

## 4. New Analyses of Published Data

The concerted effort to test the ACH is in many respects impressive. Some studies have tested very large numbers of bilinguals [19,20,21]. Sophisticated measures of DLC and other types of language switching [22,26] have been developed and used. These include entropy-based estimates of DLC that measure the number of languages used in each of several contexts [19,20,21,41,42] and the frequency judgment task used in the Hofweber studies [15,16]. The predicted EF measures have often been combined into latent variables that attenuate task impurity problems and increase confidence that performance is tapping into a domain-general control process [19,21]. Psycholinguistic approaches and theoretical perspectives have been supplemented by contributions from linguistics and sociolinguistics. Despite the high caliber of this recent research, the results are inconsistent and highlight how extremely difficult it is to get valid and reliable measures of different types of language mixing and different components of EF. Do DLC tendencies enhance EF more than SLC proclivities? Sometimes. Does DCS lead to less enhancement than DLC bilingualism? No, and maybe the reverse is true. 

To conclude this review of the research testing the ACH, we report new analyses of two of our published studies. One study [2] had 104 bilinguals and 62 monolinguals complete four commonly used nonverbal interference tasks (Simon, spatial Stroop, vertical Stroop, and flanker) with varied S-S and S-R compatibilities. No bilingual advantages were observed in any of the tasks. The participants were all San Francisco State University students. Slightly more than 60% would be considered bilingual based on a mean L2 proficiency rating of 4 or higher. There are many different language pairings represented in the sample, but the most common are English–Spanish, English–Chinese, and English–Tagalog. As shown in Table 1 and Table 2, many facets of bilingual experience were solicited and regression analyses were used to test several aspects of bilingual use (e.g., the frequency of switching within a sentence or the mean number of languages used across seven different contexts) and none of these continuous variables significantly predicted interference scores derived from four nonverbal interference tasks. A potential problem with this approach is that the ACH predicts U-shaped functions. Frequency of switching per day should enhance EF, but at sufficiently high rates it is likely to involve dense code-switching and lead to a decrease. Increases in an entropy-like measure (e.g., mean number of languages used per context) follow the same inverted-U prediction. EF performance should increase as strong SLC tendencies give way to more DLC tendencies, but dense code-switchers operating in an open control mode are likely to have large entropy scores. These nonlinear predictions cannot be properly evaluated with linear regression. Thus, a new analysis was conducted where we used the array of language measures shown in Table 1 and Table 2 to identify individua\l bilinguals whose profile closely matches “pure” cases of SLC, DLC, and DCS bilingualism. The Multilingual Naming Task (MINT) referred to in Table 1 was developed by Gollan and colleagues [43].

### 4.1. The New Analyses: Criteria for Classifying Language Groups 

In both studies, participants rated their speaking, listening, reading, and writing proficiency using the scale shown in Table 2. The scale was first used and developed by Paap and Greenberg [44]. The strategy underlying the new analysis is to use this battery of information to identify reasonably “pure” cases of SLC, DLC, and DCS bilinguals based primarily on the criteria outlined in the original ACH and compare the groups on measures of cognitive control not only to each other, but also to monolinguals.

Criteria for single-language context bilingualism. SLC bilinguals were identified with reference to five criteria. One criterion applied to all three groups was that they met a general definition of bilingualism by rating their L2 proficiency in speaking or listening as 4 or higher. The primary inclusion criterion for the SLC group was that they reported a tendency to use only one language in each of the seven contexts shown in Table 1. To qualify for this SLC group, a bilingual must have a mean number of languages per context less than 1.5. As a further check against the dual-language practice, the frequency of switching within a conversation was examined and the bilingual was disqualified if the rating of switching within a conversation was greater than 3 (meaning that they *often* or *very often* switched within conversations). Under the reasonable assumption that most conversations take place in one context, frequent switches within conversations indicate deviance from SLC bilingualism. Bilinguals were also excluded if their reported frequency of switching within sentences was greater than 3 as this implies not only speaking two languages in the same context, but also some amount of dense code-switching. It was also desirable that the SLC bilinguals currently used at least two languages and cases were excluded if the percentage of use of the most used language was greater than 90%. The means across these criteria for each group are shown in Table 3. A surprising result is that only 11 “pure” cases of SLC bilingualism were identified in our sample of 120 SFSU student bilinguals. Many bilinguals who met the primary criteria of having a low mean number of languages per context were excluded, because they reported frequent daily switching and/or frequent switching within a conversation. (Bilinguals who did not fit in any of the designated groups were assigned to a separate group of Other Bilinguals.) Other samples—even other samples of university students—may have higher proportions of SLC bilinguals. SFSU is a “commuter school” with limited resident housing and many students live at home or in local communities where their non-English language is commonly spoken. As shown in Table 3, the mean languages per context for the SLC bilinguals is substantially less than means for the DLC and DCS groups. Although they do tend to use only one language per context, they do use the minority language about 25% of the time on average. 

Criteria for dual-language context bilingualism. The primary criterion for DLC membership was using multiple languages in the same context, specifically a mean of 1.5 or greater across the seven contexts. As shown in Table 3, the DLC mean of 1.87 is well above the mean of 1.28 for the SLC group. There are more “pure” cases of DLC bilingualism (viz. 24) compared to SLC. This is good, because the clearest prediction of the ACH is that DLC tendencies should benefit cognitive control the most and, consequently, that DLC should show advantages relative to all other groups. Bilinguals were excluded from the DLC group if they used their other languages less than 20% of the time or reported daily switching only “sometimes” (Likert value of 3 or less). These two criteria seemed desirable, because benefits of switching languages are not likely to accrue if those switches only occur occasionally. Note that for the DLC group, the most used language is, on average, used only 66% of the time. Finally, if an otherwise eligible bilingual indicated that they switched within sentences “very often” (the top of the 5-point Likert scale) they became a candidate for the DCS group. Thus, the primary inclusion criterion for the DCS group was a rating of 5 on the probe for frequency of switching within sentences. Bilinguals with mean languages per context less than 1.3, with frequencies of daily switching less than 3.0, and percentages of most used language less than 20% were reassigned to the Other Bilingual group. The last three criteria were included on the assumption that dense code-switching, presumably under an open control mode, would have little effect on the development and maintenance of cognitive control if the dense code-switching was limited to only one or two contexts. Note in Table 3 that the DCS bilinguals tend to use both languages in multiple contexts (M = 1.78 languages per context) and report that they switch languages very often on a daily basis (M = 4.78 on a scale of 1 to 5). 

**Criteria for DCS.** Most important in distinguishing DCS from DLC bilingualism is the fact that all the bilinguals in the DCS group rated their within-sentence mixing as a top-of-the scale 5 (very often). They also had to have a mean of 1.3 or better on the mean number of languages spoken per context, a frequency of daily switching of at least 3, and used their other languages at least 20% of the time. 

**Criteria for monolinguals.** The monolinguals in this dataset provide another important baseline, because the ACH clearly predicts that DLC bilinguals and probably SLC bilinguals will have better cognitive control than monolinguals. As shown in the bottom rows of Table 3, there were 53 “pure” monolinguals with no exposure to a second language and 25 “other” monolinguals that had limited exposure or training in a foreign language.

### 4.2. Language Group Differences on Measures of EF from 2019 Dataset

Description of EF tasks. The tasks and factor analyses are fully described in Paap et al. [2]. Interference scores (incongruent trial RT minus congruent trial RT) were reported for a Simon task, a vertical Stroop task, a horizontal Stroop task, and a flanker task. Using an exploratory factor analysis, the first three tasks cohered into a latent variable that excluded the interference scores from the flanker task. Other things being equal, one can be more confident that composites scores from tasks that form a latent variable reflect a domain-free measure of cognitive control. Thus, our primary test of the ACH involves predicting a composite measure of interference control based on the three tasks that formed a latent variable. We also report an analysis of the flanker effect because that is the measure of cognitive control used in the two Hofweber studies [15,16] and also used in Hartanto and Yang [20,21]. 

## 5. Results

**Figure 1:**
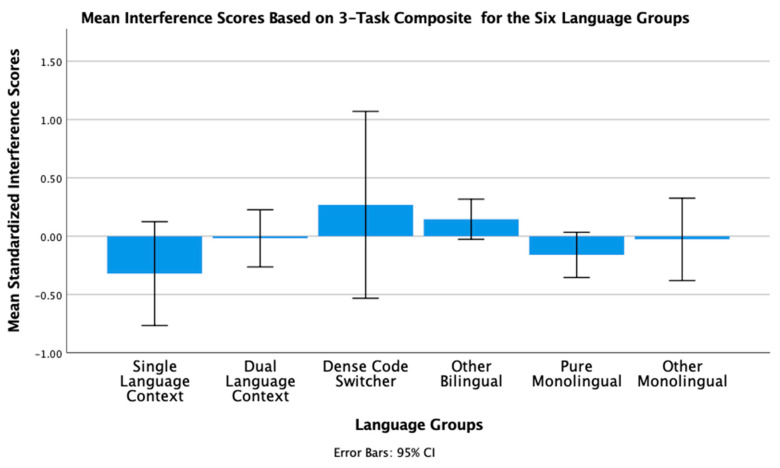
Mean interference scores (expressed as z-scores) on a composite of the Simon, vertical Stroop, and horizontal Stroop tasks for each of the six language groups.

The results of the one-way ANOVA on the z-score composite of the three tasks that formed a latent variable for the six language groups are shown in Figure 1. The results could hardly be more surprising from expectations based on the ACH. The DLC bilinguals with a mean near zero are not showing the best interference control. The DCS bilinguals that in pure form should be similar to the monolinguals show the greatest interference scores. The SLC bilinguals that should have poorer control compared to the DCS bilinguals show the smallest (best) interference scores. All of these trends aside, there are no statistically significant differences between the language groups, *F*(5, 192) = 1.72, *p* = 0.13. 

**Figure 2:**
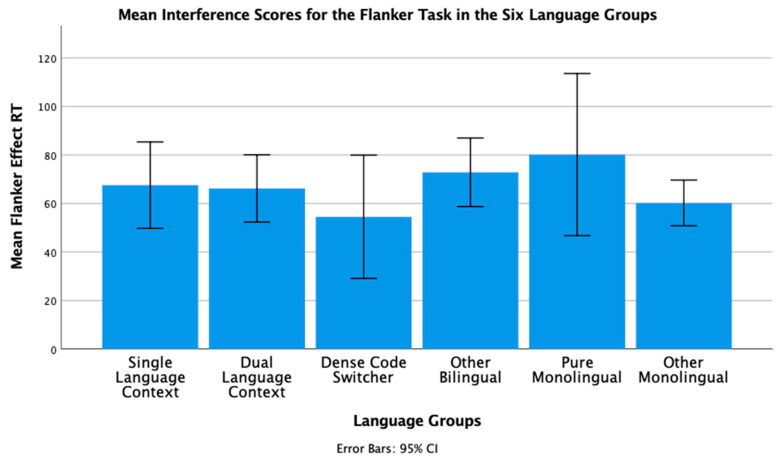
The mean flanker effect and 95% confidence intervals for each of the six language groups.

The analysis of the ANOVA on the flanker effect is shown in Figure 2. The statistical outcome is identical to that on the three-task composite, namely, no significant differences between the language groups, *F*(5, 192) = 0.37, *p* = 0.87. Again, the trends do not follow the predictions of the ACH. For example, DCS bilinguals show better interference control than either SLC or DLC bilinguals who have nearly identical means. 

### 5.1. New Analyses of 2018 Dataset

Paap and colleagues [18] tested 141 SFSU students on a battery of tasks that Bialystok [45] classified as selective-attention. These included a color–shape switching task, a conjunctive visual search task, and a spatial Stroop task. The same criteria were used to classify the participants in the six language groups. The means, standard deviations, and 95% confidence intervals for each group and for each criterion are shown in Table 4.

Inspection of Table 4 shows that this dataset, like the dataset summarized in Table 3, has more DLC bilinguals than either SLC or DCS bilinguals. Having only 10 and 11 bilinguals in the SLC and DCS groups indicates that comparisons to these group are severely underpowered and should be considered risky comparisons. However, as noted in the discussion of Table 3, a larger number (in this case n = 42) of DLC bilinguals ameliorates the situation, especially because the ACH predicts that the DLC group should be superior to all other language groups. Thus, if the DLC group is not significantly better than any of the other groups or combinations of groups then the fundamental prediction of the ACH fails. 

The Paap et al. study [18] includes several measures of cognitive control all of which Bialystok [45] has referred to as measures of selective attention. These include mixing and switching costs from a color–shape switching task (such as the one used by Hartanto and Yang [20,21]), a spatial Stroop task (often presumed to measure inhibitory control with both S-S and S-R conflict), a conjunctive visual search task [46] and a morphing ambiguous figures task [47]. The latter two were touted in the cited articles as good measures of the ability to disengage attention although the bilingual advantages were not replicated by Paap et al. [18] for either task or by Ratiu and colleagues [48] for the visual search task. As reported in detail below, there were no significant group effects in any of the seven measures of cognitive control, all exact *p* values > 0.20. 

The tasks are described in detail in Paap et al. [18]. Briefly, the spatial Stroop task presented left or right-pointing arrows displaced either to the left or right of fixation. Participants had to press a key on the left or right corresponding to the direction of the arrow and ignore the arrow’s spatial location. In the visual search task, participants were instructed to search for a blue–triangle target among distractors that differed with respect to either color (green) or shape (diamond). Trials had either 0, 5, 10, or 15 distractors. In the ambiguous figures task, a sequence of 11 drawings were presented one at a time. The drawings morphed from an unambiguous drawing of one object (e.g., a seal) to a different object (e.g., horse). Participants indicated when the figure no longer look like the start object (referred to as the AF1 dependent measures) and then when they could identify the new object (referred to as AF2). 

### 5.2. Results Based on 2018 Data

**Figure 3:**
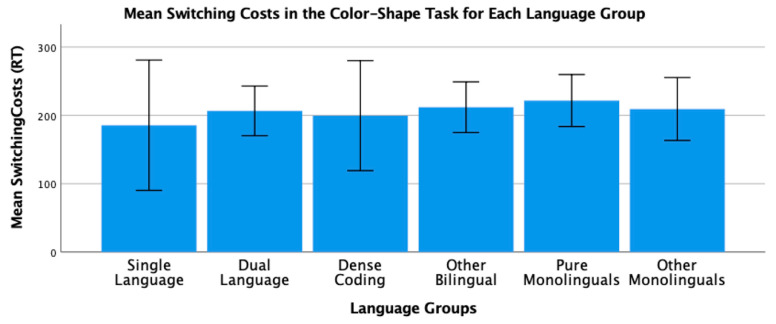
Mean switching costs (RT) across the language groups for the color–shape switching task with 95% confidence intervals.

**Figure 4:**
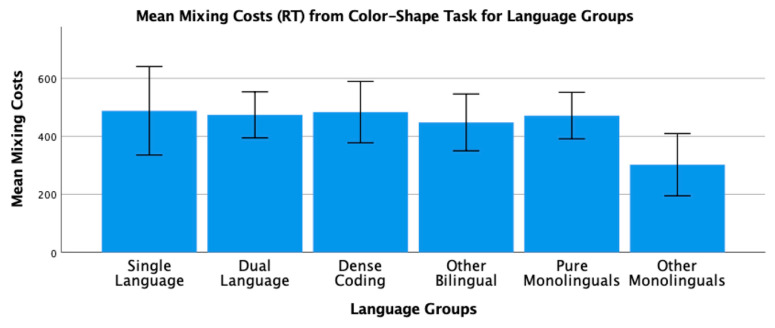
Mean mixing costs (RT) for the six language groups on the color–shape switching task with 95% confidence intervals.

Figure 3 and Figure 4 show the outcome of one-way ANOVAs for the switching costs and mixing costs derived from the color–shape switching task. The group main effect was not significant for either switching costs, *F*(5, 125) = 1.42, *p* = 0.22, or mixing costs, *F*(5, 125) = 0.214, *p* = 0.956. Inspection of the switching costs (Figure 3) shows that the critical DLC mean is very much in the middle of the pack and that it is the SLC bilinguals that show a slight tendency to have smaller switching costs. Likewise, for mixing costs (Figure 4), there is no hint that DLC bilinguals have special status, and the other monolinguals are those who show a non-significant tendency to have smaller mixing costs. 

**Figure 5:**
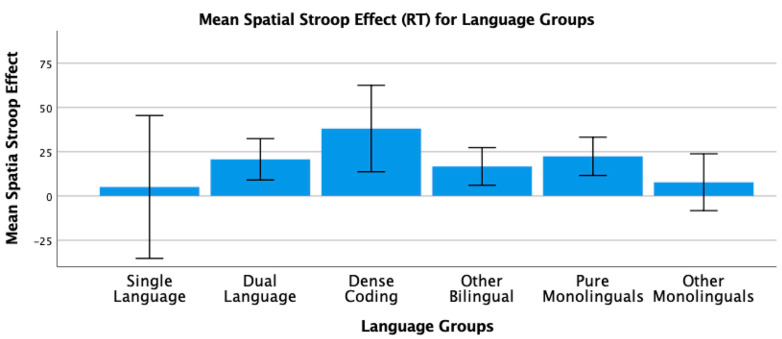
Mean spatial-Stroop effect (RT) and 95% confidence intervals for the six language groups.

Figure 5 shows the mean spatial Stroop effects. The main effect of language group was not significant, *F*(5, 130) = 1.38, *p* = 0.23. The critical DCL group once again falls in the middle of the pack. SLC bilinguals and other monolinguals are the groups showing non-significant tendencies for smaller Stroop effects.

**Figure 6:**
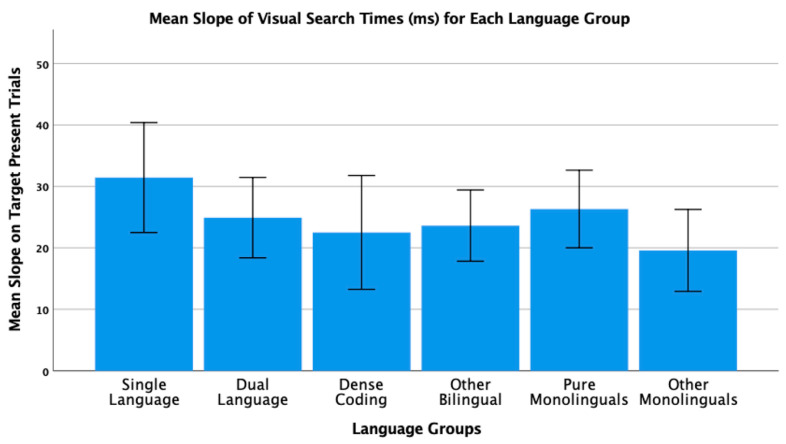
Mean slope (ms) of the visual search times on target present trials and 95% confidence intervals for the six language groups.

**Figure 7:**
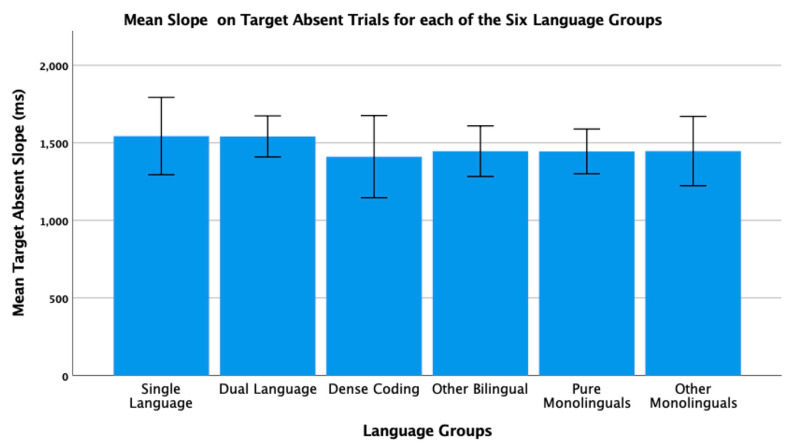
Mean slope (ms) of the visual search times on target absent trials and 95% confidence intervals for the six language groups.

As expected, visual search times as a function of the number of distractors in the original study [18] were well fit by a linear function. Thus, the slopes on target present and target absent trials provided an excellent measure of search efficiency with smaller slopes indicating more efficient search. The mean slopes for each of the six language groups are shown in Figure 6 (target present) and Figure 7 (target absent). The main effect was not significant for target present, *F*(5, 125) = 0.68, *p* = 64 or for target absent *F*(5, 125) = 0.40, *p* = 0.85. As seen with the other measures of cognitive control, the mean slope for the critical DLC bilinguals do not trend toward better performance and, in fact, when the target is absent, the DLC group has the greatest numerical slope. 

**Figure 8:**
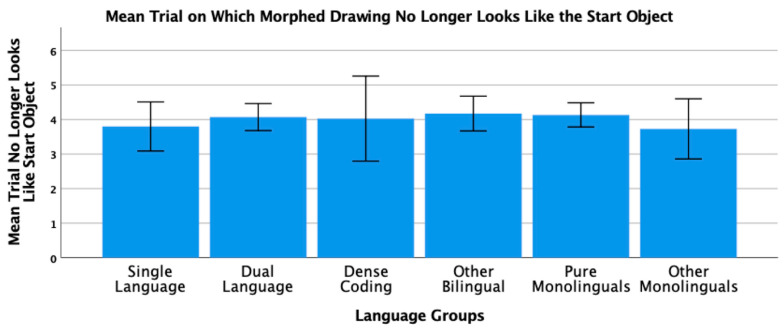
Mean trial on which the morphed drawing no longer looks like the start object and the 95% confidence intervals. This measure could reflect the ability to disengage attention or a general advantage in mental flexibility.

**Figure 9:**
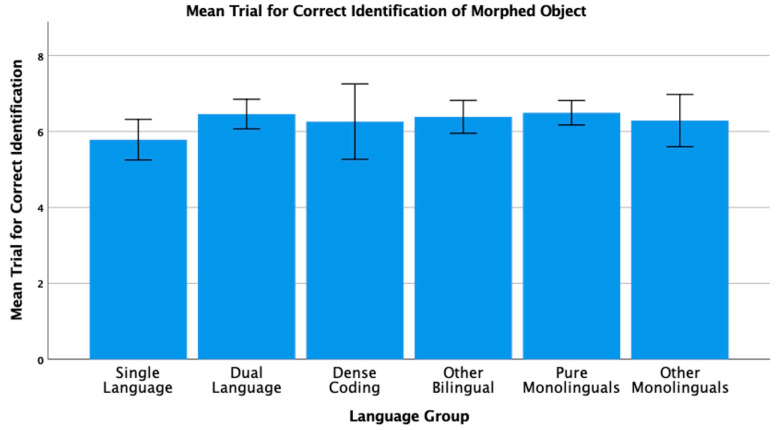
Mean trial on which the morphed drawing was correctly identified and 95% confidence intervals. This dependent variable may reflect selective attention to the critical features of the new object or a general advantage in mental flexibility.

Figure 8 and Figure 9 show the performance on the morphing ambiguous figures task. Figure 8 shows the mean trial number at which the drawing of the start object no longer looks like the start object. This measure might reflect the participant’s ability to disengage attention from the critical features of the start object. Figure 9 shows the mean trial number at which the morphed object is correctly identified. This measure might reflect the participant’s ability to selectively attend to the critical features of the new object. Neither dependent variable revealed a significant main effect of language group, *F*(5, 131) = 0.57, *p* = 0.35 for “disengagement” and *F*(5, 131) = 0.69, *p* = 0.63 for “engagement”. As shown in Figure 8, the mean for the critical DLC bilinguals is again in the middle of the pack and the other monolinguals are the ones who enjoy the numerical (but not significant) best mean. As shown in Figure 9, the mean of the critical DLC bilinguals is the highest and for this measure, lower scores are better performance. 

## 6. General Discussion

To summarize, the same criteria were used to classify participants from two datasets into six language groups. Across the two studies, there were a total of nine tests for a DLC advantage over other groups of bilinguals or monolinguals. There were no significant language-group differences for any of the nine tests. The tests included tasks favored in previous research: color–shape switching, congruency effects on nonverbal interference tasks, and tasks presumed to require selective attention that Bialystok [45] prefers over the flanker, Simon, and spatial Stroop tasks. One test was based on a composite of three interference tasks that cohere into a latent variable and consequently shares the latent-variable approach used in the excellent studies by Hartanto and Yang [21] and Kalamala et al. [19]. Although we assessed the switching component only with the color–shape task, we have shown that this exact task coheres into a latent variable with two other cued switching tasks [49]. We also included two groups of monolinguals, a comparison that is often missing, but is really needed when there is a DLC advantage over another bilingual group. For example, although Hartanto and Yang showed a switching cost advantage for DLC tendencies over SLC tendencies it is unknown if DLC bilingualism would also be superior to monolingualism. If that hypothetical difference was not significant, then one would question whether the advantage of DLC over SLC bilingualism was due to different types or amounts of bilingual language control.

Although there is some evidence supporting the predictions derived from the ACH, the primary prediction that control processes exercised by DCL bilinguals should enhance cognitive control compared to SLC bilinguals or monolinguals received inconsistent and weak support. With regard to advantages on switching, the supporting evidence [20,21] is compromised by the lack of a monolingual baseline, the null results for switch costs in Lai and O’Brien [22] and the switching-cost results shown in Figure 3. With regard to advantages in interference control or response inhibition, these are strongly challenged by the null results for the latent variable of response inhibition in Kalamala et al., [19] the interference scores for a composite variable (Figure 1), the flanker effect (Figure 2), and the spatial Stroop effect (Figure 5). An even greater challenge to DCL superiority is presented in the Hofweber studies [15,16] showing that dense-code switching based on word mixing (also known as congruent lexicalization) is associated with the smallest interference scores. 

Our reanalysis aimed at identifying relatively pure cases of the three interactional contexts specified by the ACH is constrained by the inherent tradeoff between purity and sample size. Strict criteria were adopted under the belief that purity was more important than size, because all the studies based on continuous measures of DLC intensity or propensity would inherently include all the bilinguals, including those in the vast middle range, that were likely to contribute little to teasing apart the relative demands of compartmentalization versus high entropy. One can view the studies that use continuous measures (and consequently all of the bilinguals) as complementary to those that seek to identify subsets of relatively homogeneous and contrasting subsets. 

### Limitations

Small N for SLC and DCS groups. To review, the reanalyses identified six language groups: DLC, SLC, DCS, other bilinguals, pure monolinguals, and other monolinguals. Across the two datasets, the SLC and DCS groups consisted of 10 ± 1 bilinguals. Clearly, the estimates of mean EF performance for these groups lacked the desired precision. Although the DLC groups of n = 42 and n = 24 are somewhat under-powered, these are quite typical (see Figure 4 of Paap, et al. [14]) and given the additional criteria used to screen out atypical bilinguals (e.g., not active bilinguals, have contradictory responses, etc.) who otherwise have high DLC scores our DLC groups are quite homogeneous. That was the point of trying to identify “pure” cases. Finally, this is the very group that the ACH predicts should be superior to all six of the other language groups, but it is never the best group numerically and it is never significantly better than any of the other five groups considered separately. Thus, there are no hints in the trends that greater power would uncover DLC advantages. 

Measure of language use. The least controversial conclusion drawn in this review is that it is extremely difficult to devise valid and reliable measures of different modes of bilingual language control and of EF. The criteria used to identify SLC, DLC, and DCS in the supplementary analyses reported in Figure 1, Figure 2, Figure 3, Figure 4, Figure 5, Figure 6, Figure 7, Figure 8 and Figure 9 are intuitively appealing but may not be adequate. The most critical criterion for distinguishing SLC from DLC was the mean number of languages used across seven contexts. Unlike the entropy measures used by Gullifer and Titone [41,42] and by Kalamala et al. [19] participants were not asked to make fine-grain judgments about the percentage of time using each language in each context. Because our data was collected before becoming aware of the entropy measures, we had doubted (and remain skeptical) that bilinguals could accurately judge these percentages and maintain the motivation to do so across seven contexts. Thus, some studies have relied on nuanced judgments in each of four contexts, while our studies relied on simpler judgments in each of seven contexts. The frequency judgment task used in the Hofweber studies [15,16] may be a good way of distinguishing between dense code-switching that features alternations versus word insertions versus congruent lexicalization, but it requires a new set of materials for each language pair. It may be worthwhile to test these various approaches with a corpus obtained from bilinguals willing to have a large sample of their natural conversations recorded.

The frequent failures of the far-transfer predictions derived from the ACH (in both the reviewed literature and in our new re-analyses) may be caused more by the fraught measures of interactional context than problems in the veracity or underlying logic of the adaptive control model itself. It is surprising that there is so little discussion of the extent to which bilinguals can judge the relative frequencies needed to calculate entropy or DLC tendencies. These measures rely on the assumption that most participants carefully read the probes, interrogate their episodic memories for interactional events matching the probe, and derive an accurate average of the frequency that they switched languages in each context. This seems to merit, at a minimum, some type of validity check. A recent effort using ecological momentary assessment [50] to do just that concluded that retrospective self-reports of language switching may lack convergent validity. Another problem with the typical self-reports is that many bilinguals hold negative attitudes towards code-switching and therefore tend to under-report code-switching practices such as those used to define DCS [51]. 

**How much does bilingual language control rely on domain-general EF?** Hofweber et al. (2016) do not say much about the “local inhibition” exercised during congruent lexicalization and why this should be considered, at least to some extent, the same inhibitory-control mechanism that presumably affects the magnitude of the flanker effect. However, the idea of dual control modes was further developed in Hofweber, et al.’s [16] study, in which they explain how these notions may map onto proactive and reactive control. In the four years since Hofweber earlier study [15], concerns about the existence of a domain-free inhibitory-control ability have become acute [40] because the interference control in the flanker, Simon, and spatial Stroop tasks appears to be task specific. Alarmingly, back in 2010, Salthouse [52] reported that the letter and arrow instantiations of the flanker task do not correlate. Paap et al. [2] reported that two versions of the Simon task and a spatial Stroop task cohered into a latent variable, but that an arrow-version of the flanker task did not load on this variable. A study by Rey-Mermet and colleagues [53] used six tasks assumed to reflect *Inhibition of Prepotent Responses* and five assumed to reflect *Resistant to Distraction*. They concluded that nonverbal tests used to assess “inhibition” do not measure a common, underlying construct, but instead, measure the highly task-specific ability to resolve the interference arising in each task. For them, the *“… inevitable implication is that studies using a single laboratory paradigm for assessing or investigating inhibition do not warrant generalization beyond the specific paradigm studied”,* (p. 515).

Similarly, Paap et al. [40] recommended that we should stop evaluating the consequences of bilingualism (or other special experiences) on EF by using single tasks, especially the flanker task, because these reflect mostly task-specific control mechanisms. Indeed, one reason why it may be so difficult to consistently produce significant differences between types of bilinguals or between bilinguals and monolinguals is that bilingual language control is encapsulated within the language processing system [2] and, consequently, is different from the task-specific mechanisms used in the common measures of EF.

Blanco-Elorrieta and Pylkkanen [54] have made a similar argument about switching. They reviewed a body of work showing that when bilinguals switch languages voluntarily, both the behavioral switch costs and the activation of brain regions associated with cognitive control are greatly reduced or eliminated. This pattern suggests that switching languages is not inherently effortful, does not usually require top–down control, and therefore, bilingual advantages in general switching costs may be limited to bilinguals who frequently switch languages based on unpredictable external constraints. Bilinguals in this category may be unicorns.

## 7. Conclusions

In conclusion, the evidence supporting the predictions derived from ACH for far transfer to nonverbal measures of EF is not compelling, as there is no coherent pattern to when predictions succeed and when they fail, and failures far outnumber successes. This is true even if one attends only to the most straightforward prediction that high DLC tendencies should lead to better inhibitory control and switching ability compared to all other language groups. Despite the insight and care that has been applied to theorizing and measuring different aspects of bilingual language control, future research must improve on this foundation. At this time, it is impossible to determine if the sporadic support of the ACH is genuine or the product of an idiosyncratic combination of bilinguals and EF tests that resonates with tendencies toward confirmation bias [31] and overfitting of the data [42]. Finally, it is important to acknowledge that the value and scope of the ACH in guiding research and understanding of bilingual language control go far beyond the predictions that it inspired about bilingual advantages in EF. This study focused only on its contribution to the latter.

## Figures and Tables

**Table 1:** Information Solicited about Language Use and Proficiency.

Probe for Indicated Attribute	Response
Languages: *We would like to know about the languages you currently speak and understand (even if those language abilities are very limited). Ignoring your ability to read or write, which languages do you speak and understand? List from most understood to least understood and leave blank if not applicable.*	participantenters list of languages
Proficiency: *Rate your ability to speak English.* {Repeated for *understand spoken English*, *read English*, *write* *English* and then for all other listed languages.}	Likert 1 to 7 (See Table 2)
L1 Proficiency: Mean of speaking and listening for highest rated language.	mean
L2 Proficiency: Mean of speaking and listening for second-highest rated language.	mean
Language Balance: The L2/L1 Proficiency ratio.	division
Changes in Proficiency: *Were you ever more proficient in Spanish than you are now?* (Repeated for up to 3 languages.) *Please use the following rating scale to rate what you highest level of proficiency was.*	Likert 1 to 7
English Productive Vocabulary: Multilingual Naming Task (MINT)	total correct
Percentage of Use: *What percentage of the time do you use Spanish?* {Repeated for each language with constraint that percentages sum to 100.}	number entry
Percent Use of Most-Used Language	computed
Native Languages. *Native languages are those you were exposed to by family or caretakers during the first few months of life. Check all languages you regularly heard during the first few months to the best of your knowledge.*	check list
Bilingual Community: *Currently, how often do you talk to others who speak the same two languages you do?*	Likert 1 (never) to 5 (very often)
Daily Switching: *Some bilinguals switch from one language to another many times every day because they talk to others who speak the same languages. Others rarely switch because they use one language in some places and the other language in other places. On a typical day how often do you switch?*	Likert 1 (never) to 5 (very often)
Switching Within a Conversation: *When speaking with others who know the same language a conversation might start in one language and then switch to another. In a typical conversation, how often do you switch?*	Likert 1 (never) to 5 (very often)
Mixing Within a Sentence: *When producing a sentence in one language bilinguals sometimes replace a word or two with its translation equivalent in their other language. How often do you do this?*	Likert 1 (never) to 5 (very often)
Gap Insertions: *A single word from the other language might be inserted into a sentence because the speaker does not know (or can’t think of) the word in the intended language. How often does this happen to you?*	Likert 1 (never) to 5 (very often)
Precision Insertions: *A single word from the other language might be inserted into a sentence because the word means something slightly different and better expresses the intended meaning. How often do you do this?*	Likert 1 (never) to 5 (very often)
Where You Live: *Check all languages you speak where you currently live (e.g., dorm, apartment, house, etc.).*	check list
With Family: *Check all languages you use when speaking with your family (face-to-face or phone).*	check list
With Friends: *Check all languages you use when speaking with your friend (face-to-face or phone).*	check list
When at Work: *Check all languages you use when you are at work. If not employed leave this column blank.*	check list
When at School: *Check all languages you use when you are at school (in class/socializing on campus. If you are currently not a student leave this column blank.*	check list
In Local Community: *Check all languages you use when you are in your local community (e.g., clerks, cashiers, food service, transportation services, health-care services, government services, etc.)*	check list
Media: *Check all* languages *you listen to in the entertainment media (e.g., music, TV, streaming video, internet)*	check list
Mean Languages Per Context Mean number of languages used per context	computed
Years Resided in USA: How long have you lived in the United States?	entry

**Table 2:** Rating Scale Used to Solicit Proficiency Ratings for Speaking, Listening, Writing, and Reading.

Rating Value	Rating Label
1	Beginner: Know some words and basic grammar
2	Advanced Beginner—Can converse with a native speaker only on some topics and with quite a bit of difficulty
3	Intermediate—Can converse with a native speaker on most everyday topics, but with some difficulty
4	Advanced Intermediate—Can converse with little difficulty with a native speaker on most everyday topics, but with less fluency than a native speaker
5	Near Fluency—Almost as good as a typical native speaker on both everyday topics and specialized topics I know about
6	Fluent—As good as a typical native speaker
7	Super Fluency—Better than a typical native speaker.

**Table 3:** Descriptive Statistics and 95% Confidence Intervals for Criterial Measures for Each Language Group.

Language Attribute	N	Mean (SE)	Lower Bound	Upper Bound
Group				
Mean Languages Per Context				
SLC	11	1.28 (0.20)	1.14	1.42
DLC	24	1.87 (0.06)	1.75	2.00
DCS	9	1.78 (0.36)	1.50	2.05
Other Bilinguals	76	1.48 (0.37)	1.40	1.57
Daily Switching				
SLC	11	2.64 (0.67)	2.18	3.09
DLC	24	4.21 (0.59)	3.96	4.46
DCS	9	4.78 (0.44)	4.44	5.12
Other Bilinguals	74	1.18 (0.14)	2.92	3.46
Switching Within Sentences				
SLC	11	2.91 (0.30)	2.71	3.11
DLC	24	2.83 (0.08)	2.67	2.99
DCS	9	5.00 (0.00)	-	-
Other Bilinguals	73	0.95 (0.11)	2.96	3.40
Switching Within Conversations				
SLC	11	2.64 (0.50)	2.30	2.98
DLC	24	3.08 (0.83)	2.73	3.43
DCS	9	4.44 (0.34)	3.67	5.22
Other Bilinguals	74	2.99 (1.04)	2.75	3.23
% of Most Used Language				
SLC	11	74.5 (14.4)	64.9	84.2
DLC	24	60.6 (13.4)	55.0	66.3
DCS	9	59.3 (10.2)	51.5	67.2
Other Bilinguals	76	78.1 (16.7)	74.3	82.0
L2 Proficiency				
SLC	11	5.04 (0.82)	4.49	5.60
DLC	24	5.77 (0.79)	5.44	6.11
DCS	9	5.28 (1.12)	4.42	6.14
Other Bilinguals	76	4.96 (1.17)	4.69	5.23
L2/L1 Proficiency Ratio				
SLC	11	0.82 (0.15)	0.725	0.924
DLC	24	0.87 (0.12)	0.824	0.921
DCS	9	0.83 (0.16)	0.707	0.958
Other Bilingual	76	0.76 (0.17)	0.723	0.801
L1 Proficiency				
SLC	11	6.2 (0.72)	5.70	6.66
DLC	24	6.6 (0.42)	6.45	6.80
DCS	9	6.3 (0.43)	6.00	6.67
Other Bilinguals	76	6.5 (0.53)	6.39	6.63
Pure Monolinguals	53	6.5 (0.46)	6.38	6.64
Other Monolinguals	25	6.8 (0.39)	6.60	6.92

**Table 4:** Descriptive Statistics and 95% Confidence Intervals for Criterial Measures for each Language Group.

Language Attribute	N	Mean (SE)	Lower Bound	Upper Bound
Group				
Mean Languages Per Context				
SLC	10	1.22 (0.10)	1.15	1.30
DLC	42	1.72 (0.27)	1.64	1.80
DCS	11	1.65 (0.25)	1.48	1.82
Other Bilinguals	29	1.36 (0.27)	1.26	1.46
Daily Switching				
SLC	10	2.60 (0.84)	2.00	3.20
DLC	42	3.81 (0.89)	3.53	4.09
DCS	11	4.64 (0.67)	4.18	5.09
Other Bilinguals	29	2.83 (1.07)	2.42	3.24
Switching Within Sentences				
SLC	10	2.90 (0.88)	2.27	3.53
DLC	42	3.38 (0.73)	3.15	3.61
DCS	11	5.00 (0.00)	5.00	5.00
Other Bilinguals	29	2.93 (1.00)	2.55	3.31
Switching Within Conversations				
SLC	10	2.60 (0.70)	2.10	3.10
DLC	42	3.74 (1.06)	3.41	4.07
DCS	11	4.36 (0.81)	3.82	4.91
Other Bilinguals	29	2.76 (1.02)	2.37	3.15
% of Most Used Language				
SLC	10	74.0 (9.7)	67.1	80.9
DLC	42	66.7 (13.6)	62.5	71.0
DCS	11	59.4 (26.3)	48.6	70.3
Other Bilinguals	27	83.0 (14.4)	77.5	88.5
L2 Proficiency				
SLC	10	4.25 (1.06)	3.49	5.01
DLC	42	5.68 (0.96)	5.38	5.98
DCS	11	5.73 (1.03)	5.03	6.42
Other Bilinguals	27	3.84 (1.35)	3.33	4.36
L2/L1 Proficiency Ratio				
SLC	10	0.66 (0.18)	0.536	0.792
DLC	42	0.90 (0.17)	0.851	0.957
DCS	11	0.87 (0.17)	0.757	0.989
Other Bilingual	29	0.62 (0.27)	0.517	0.723
L1 Proficiency				
SLC	10	6.5 (0.49)	6.09	6.81
DLC	42	6.3 (0.58)	6.15	6.51
DCS	11	6.6 (0.44)	6.30	6.88
Other Bilinguals	29	6.4 (0.77)	6.10	6.69
Pure Monolinguals	32	6.5 (0.20)	6.41	6.56
Other Monolinguals	25	6.4 (0.69)	6.02	6.78

## Data Availability

The SPSS datafiles used for the reanalyses are available upon request from the authors.
